# Pro inflammatory stimuli enhance the immunosuppressive functions of adipose mesenchymal stem cells-derived exosomes

**DOI:** 10.1038/s41598-018-31707-9

**Published:** 2018-09-06

**Authors:** Rossana Domenis, Adriana Cifù, Sara Quaglia, Cinzia Pistis, Massimo Moretti, Annalisa Vicario, Pier Camillo Parodi, Martina Fabris, Kayvan R. Niazi, Patrick Soon-Shiong, Francesco Curcio

**Affiliations:** 1 Dipartimento di Area Medica, Università degli Studi di Udine, Udine, 33100 Italy; 2 VivaBioCell S.p.A., Udine, 33100 Italy; 3 Istituto di Patologia Clinica, Azienda Sanitaria Universitaria Integrata di Udine, Udine, 33100 Italy; 4 Clinica di Chirurgia Plastica, Azienda Sanitaria Universitaria Integrata di Udine, Udine, 33100 Italy; 5 NantBioScience, Inc Culver City, Culver City, CA 90232 USA

## Abstract

The predominant mechanism by which adipose mesenchymal stem cells (AMSCs) participate to tissue repair is through a paracrine activity and their communication with the inflammatory microenvironment is essential part of this process. This hypothesis has been strengthened by the recent discovery that stem cells release not only soluble factors but also extracellular vesicles, which elicit similar biological activity to the stem cells themselves. We demonstrated that the treatment with inflammatory cytokines increases the immunosuppressive and anti-inflammatory potential of AMSCs-derived exosomes, which acquire the ability to shift macrophages from M1 to M2 phenotype by shuttling miRNA regulating macrophages polarization. This suggests that the immunomodulatory properties of AMSCs-derived exosomes may be not constitutive, but are instead induced by the inflammatory microenvironment.

## Introduction

Mesenchymal stem cells (MSCs) could be easily isolated from adult tissue and, by virtue of their large *ex vivo* expansion capacity, multipotency and immunosuppressive activity, have become a popular experimental therapeutic agent in many human diseases. Of note, the capacity of MSCs to secrete a variety of trophic factors with different functions has motivated the interest of evaluating their local or systemic injection to stimulate tissue repair in different pathologies^1^.

The anti-inflammatory properties of MSCs has been linked to their immunosuppressive potential^2^.

MSCs are able to regulate the immune response suppressing T-cell proliferation, cytokines secretion and cytotoxicity, regulating the functions of regulatory T cells, inhibiting proliferation of B cells, and maturation, activation and antigen presentation of dendritic cells and interleukin-2 (IL-2)-induced natural killer cell activation (for review see^3^).

Intriguingly, it has been also proposed that MSCs immunosuppressive ability is not constitutive; instead, it is induced by inflammatory cytokines, such as those present in the inflammatory microenvironment^4^. Exposure of MSCs to IFNγ increases the immune suppressive activity by stimulating the production of inhibitors of inflammation, such as indoleamine-pyrrole 2,3-dioxygenase (IDO)^5^, Factor H^6^, prostaglandin E_2_ (PGE_2_)^7^, TGFβ and HGF^8^. Another inflammatory mediator known to induce regenerative activities in MSCs is the macrophage-derived cytokine TNFα, which endowed the cells with superior angiogenetic activity *in vitro* as well as *in vivo* in an animal model of critical limb ischemia^9^.

Although the underlying mechanisms of MSCs immunomodulation is still to be elucidated, they are likely mediated both by soluble factors^2^ and cell-contact-dependent mechanisms^3^.

Recently, it has been proposed that the predominant way by which MSCs participate to tissue repair is through a paracrine activity^10^: in animal models, conditioned media reproduces benefits reported with the direct injection of MSCs^11^. The paracrine hypothesis has been strengthened by the recent discovery that stem cells release not only soluble factors but also extracellular vesicles, which elicit similar biological activity to the stem cells themselves^12^. The most prominent of the extracellular vesicles are exosomes, a class of small (40–100 nm) vesicles derived from endosomes through the invagination of the endosomal membrane. MSCs-derived exosomes release and transfer proteins, bio-active lipids and nucleic acid cargo, thus being able to induce phenotypic and functional changes in the recipient cells and promote the activation of regenerative programs^13,14^.

The regenerative activities of MSCs-derived exosomes are a subject of ongoing investigation. MSCs-derived exosomes are thought to have similar functions to MSCs such as repairing and regeneration of injured tissues, but little is known about the immunomodulatory effect of these vesicles and results reported in literature are discordant.

It has been demonstrated that MSCs-derived exosomes are able to induce anti-inflammatory IL-10 and TGF-β transcripts and attenuate pro-inflammatory IL-1β, IL-6, TNF-α and IL12P40 transcripts in THP-1 monocytic cell line^15^ and have an inhibitory effect in the differentiation and activation of T cells^16^. Moreover, MSCs-derived exosomes extracted from healthy donors’ bone marrow are able to suppress PBMCs secretion of pro-inflammatory factors such as TNF-α and IL-1β and increase the concentration of the anti-inflammatory cytokine TGF-β. In addition, these vesicles may induce conversion of T helper type 1 (Th1) into T helper type 2 (Th2) cells and reduce potential of T cells to differentiate into IL-17-producing effector T cells (Th17)^17^. On the other hands, it has been also reported that exosomes secreted by MSCs fail to suppress lymphocytes proliferation^18^, hypothesizing that cell-cell contact plays an important role on their immunosuppressive potential.

In this study, we investigated the immunomodulatory properties of exosomes released from adipose mesenchymal stem cells (AMSCs) under stimulation with IFNγ and TNFα inflammatory cytokines in order to evaluate the influence of inflammatory environment on exosomes production and functions.

## Results

### Effect of the stimulation with cytokines IFNγ and TNFα on AMSCs

**Figure 1:**
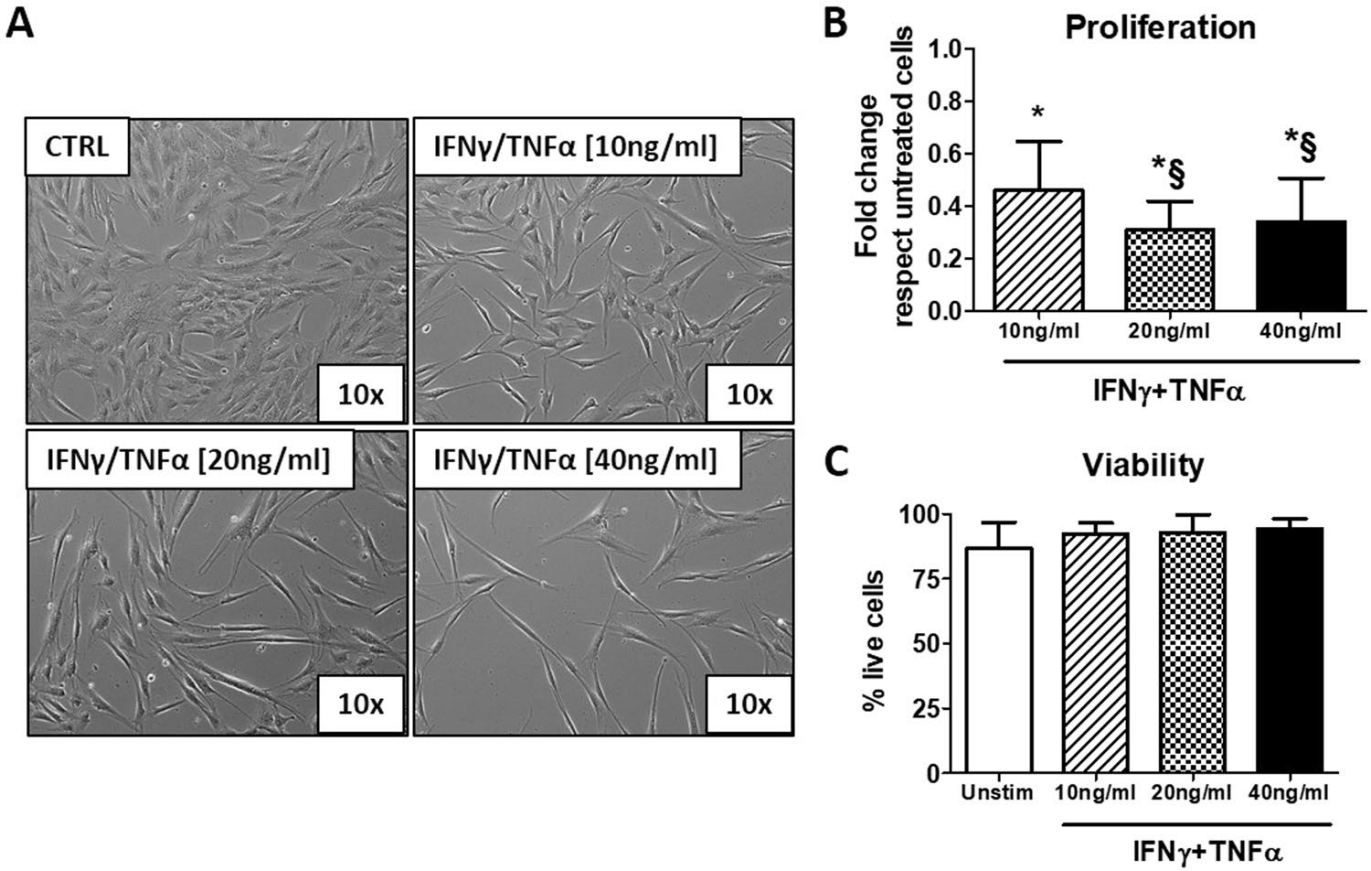
Stimulation with IFNγ/TNFα mixture induces morphological changes and inhibits proliferation of AMSCs. (**A**) Representative phase contrast images (10x magnification) of AMSCs incubated with IFNγ/TNFα at concentration of 10, 20 and 40 ng/ml for 48 hours. (**B**–**C**) Cell proliferation and viability were determined by trypan blue exclusion assay. Columns, mean; bars, SD * significant difference from unstimulated cells; § significant difference from treatment with IFNγ/TNFα at concentration of 10 ng/ml, *P* < 0.05.

AMSCs isolated by adipose tissues were tested using specific surface markers by flow cytometry: the tested AMSCs were almost completely negative for the hematopoietic markers (CD34 and CD45) and >95% positive for the mesenchymal stem cells markers (CD29, CD73, CD90 and CD105) (Supplementary Figure 1). To determine whether inflammatory stimuli may affect morphology and viability of AMSCs, cells were cultured in the presence of IFNγ/TNFα at concentration of 10, 20 and 40 ng/ml for 48 hours. We observed that the treatment with cytokines induced morphological changes, since the cells become more elongated and are characterized by an irregular shape (Fig. 1A). The incubation with cytokines decreased AMSCs proliferation in a concentration-dependent manner (Fig. 1B), while cells viability was not affected (Fig. 1C).

**Figure 2:**
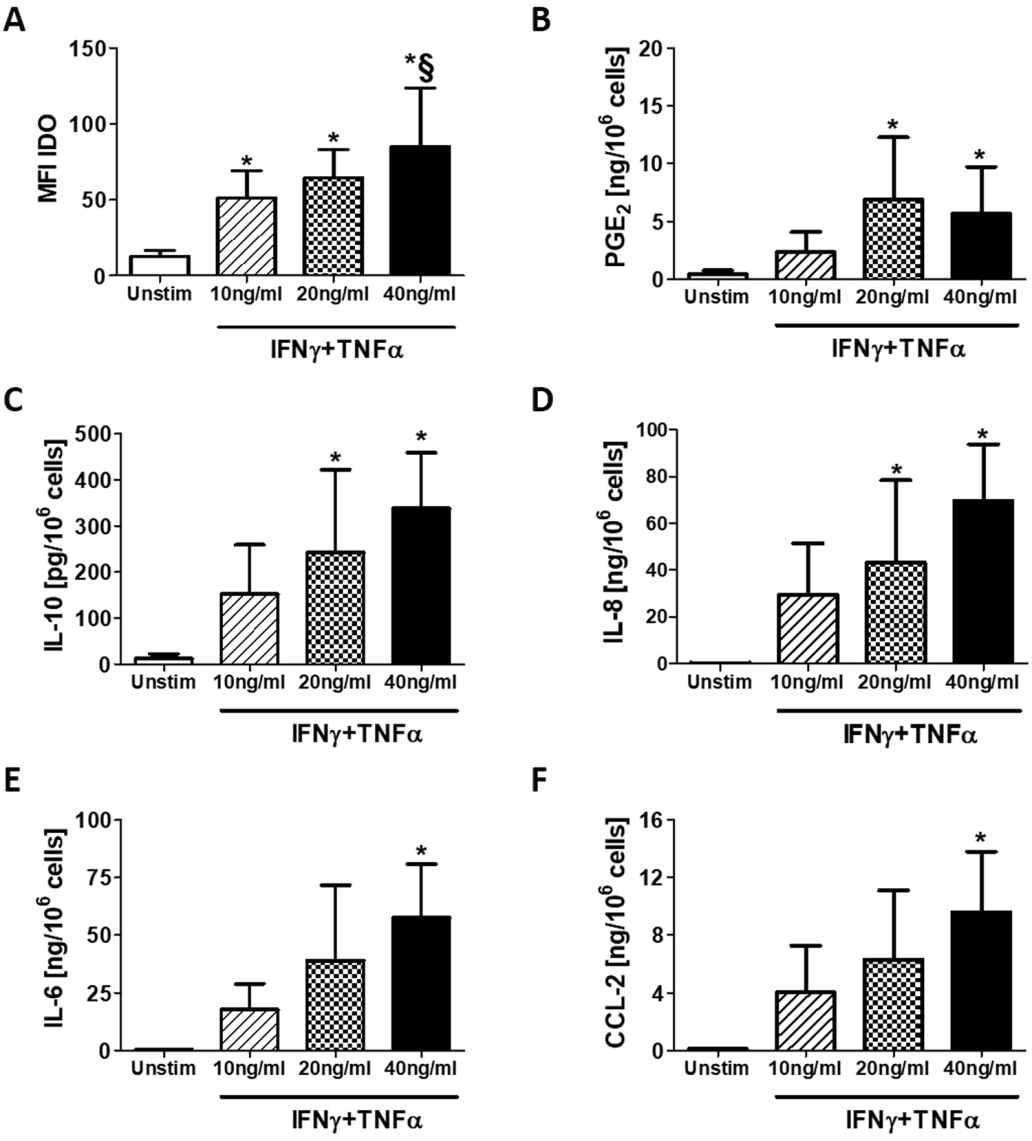
Stimulation with IFNγ/TNFα mixture induce the expression of immunosuppressive factors, cytokines and chemokines in AMSCs. AMSCs were treated with IFNγ/TNFα at concentration of 10, 20 and 40 ng/ml for 48 h. Expression of IDO was determined by flow cytometry while PGE_2_ and cytokines/chemokines production was measured in supernatants by ELISA kit. Columns, mean; bars, SD, * significant difference from unstimulated cells, § significant difference from treatment with IFNγ/TNFα at concentration of 10 ng/ml, *P* < 0.05.

We next examined the effect of inflammatory stimuli on the release of immunosuppressive factors and cytokines/chemokines by AMSCs. Treatment with IFNγ/TNFα increased the expression of the enzyme IDO in a concentration-dependent manner (Fig. 2A), while the release of PGE_2_ (Fig. 2B), IL-10 (Fig. 2C) and IL-8 (Fig. 2D) was significantly induced only after treatment with at least 20 ng/ml of the IFNγ/TNFα mixture. Finally, IL-6 (Fig. 2E) and CCL-2 (Fig. 2F) upregulation was significant only after treatment with 40 ng/ml of IFNγ/TNFα.

### Characterization of AMSCs derived-exosomes after stimulation by pro-inflammatory cytokines

AMSCs were pre-treated with IFNγ/TNFα at increasing concentration (10, 20 and 40 ng/ml), then an enriched fraction of exosomes was obtained from the supernatants using the Exoquick polymer-based strategy.

**Figure 3:**
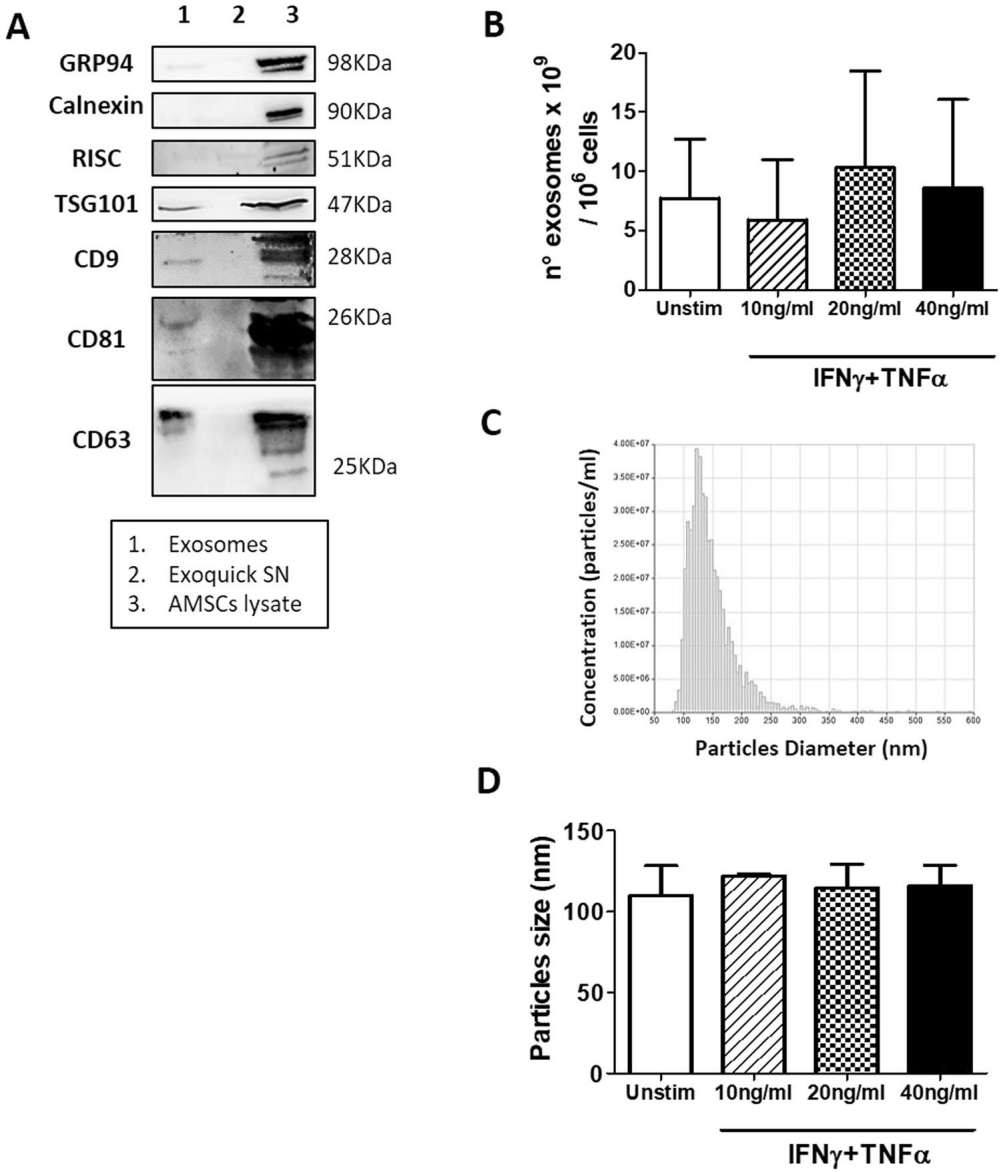
Characterization of AMSCs derived–exosomes. (**A**) Immunoblotting of AMSCs-derived exosomes, Exoquick-derived supernatants (SN) and AMSCs lysate for CD9, CD63, CD81 and TSG101 exosomal protein and Calnexin, GRP94 and RISC contaminants (cropped images – uncropped originals available in Supplementary Figure 2). (**B**) The concentration of exosomes was quantified measuring the enzymatic activity of the exosomal AChE enzyme by Exocet kit. Particles size were quantified by qNano system (**D**) and a representative graph of frequency size distribution is shown (**C**). Columns, mean; bars, SD.

As shown in Fig. 3A, AMSCs-derived exosomes and AMSCs lysates expressed the specific exosomal markers CD9, CD63, CD81 and TSG101, while no signal was observed for Exoquick-derived supernatant samples, that were used as negative control. In order to evaluate the impurities in our exosome preparations, we also evaluated the expression of proteins associated with subcellular compartments, which are supposed to be absent or under-represented in exosomes. Our data showed lack of calnexin (endoplasmic reticulum protein) and RISC complex (nucleus protein) in exosome fraction, indicating successful enrichment. We also reported a faint band for GRP94 (endoplasmic reticulum protein) in the exosomal fraction probably due to a slight contamination by apoptotic bodies.

The concentration of AMSCs-derived exosomes was determined measuring the activity of AChE by Exocet kit. As reported in Fig. 3B, the mean concentration of exosomes released by untreated cells was 7.6 ± 2.6 × 10^9^ per million of producing cells. Treatment with cytokines did not influence the number of exosomes released by AMSCs.

Finally, the average size of the collected vesicles, determined by qNano technology, was 115 ± 11.5 nm, in range with exosomes proper size, and was not influenced by AMSCs cytokines treatment (Fig. 3D).

### Exosomes derived from AMSCs pre-activated with pro-inflammatory cytokines induce an anti-inflammatory M2 phenotype reverting M1 differentiation

**Figure 4:**
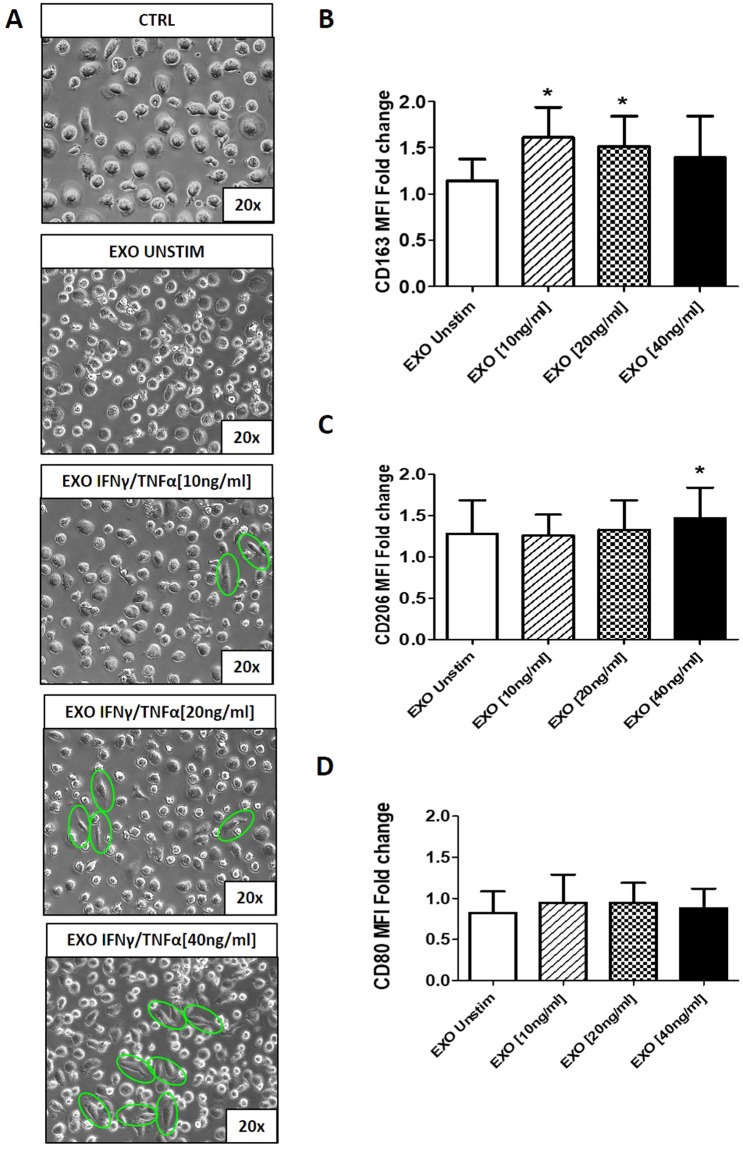
Exosomes derived from AMSCs pre-activated with pro-inflammatory cytokines induce an anti-inflammatory M2 phenotype reverting M1 differentiation. (**A**) Representative phase contrast microscopic images (20x magnification) of monocytes differentiated into macrophages in presence of GM-CSF alone (CTRL) or in combination with exosomes isolated from the supernatants of unstimulated (EXO UNSTIM) or cytokines-activated (EXO IFNγ/TNFα 10, 20 and 40 ng/ml) AMSCs. The green circles evidence cells with elongated, spindle-like morphology, a typical feature of M2 macrophages. Flow cytometry analysis of cell surface molecules CD163 (**B**) CD206 (**C**) and CD80 (**D**) on macrophages. The levels of expression are presented as median fluorescent intensity (MFI) fold change respect untreated cells. Columns, mean; bars, SD, * significant difference from unstimulated cells, *P* < 0.05.

To examine the ability of AMSCs-derived exosomes in inducing anti-inflammatory phenotype in macrophages, CD14+ monocytes isolated from PBMCs of blood donors were induced to differentiate into M1 macrophages with GM-CSF in presence of exosomes isolated from supernatants of AMSCs pre-activated with IFNγ/TNFα. As shown in Fig. 4A, at day 9, control monocytes gave rise to “fried egg-shaped” morphology, a typical feature of M1-like macrophages. When monocytes were differentiated in the presence of exosomes obtained from pre-activated AMSCs, some cells displayed an elongated, spindle-like morphology, a typical feature of M2 macrophages^19^. The effect is particularly evident in monocytes incubated with exosomes isolated from AMSCs pre-activated with 40 ng/ml of IFNγ/TNFα. Indeed, compared to untreated M1-like macrophages, only exosomes isolated from pre-activated AMSCs are able to upregulate the expression of the M2 macrophage marker CD163 (Fig. 4B). With regard to CD206 expression, it became significant only after treatment with exosomes produced by pre-activated cells with 40 ng/ml of IFNγ/TNFα (Fig. 4C). In contrast, the expression of the M1 macrophage marker CD80 did not change significantly in the presence of AMSCs-derived exosomes (Fig. 4D).

In order to evaluate a possible contamination of IFNγ and TNFα in exosome preparations, the culture medium supplemented with cytokines at different concentrations (10, 20 and 40 ng/ml) was treated with Exoquick and concentration of IFNγ and TNFα was measured by magnetic beads-based multiplex assay.

We found slightest amounts of IFNγ (0.096 ± 0.003 pg/ml) and TNFα (0.039 ± 0.001 pg/ml), which are minimal compared to those used in literature to stimulate monocytes or macrophages. Moreover, we evaluated the potential effect of these contaminants during monocytes differentiation, finding that they did not influence macrophages polarization in the expression of CD80 (Supplementary Figure 3A) and CD163 (Supplementary Figure 3B).

### Exosomes derived from AMSCs pre-activated with inflammatory cytokines contained miRNAs involved in M2 macrophages polarization

**Figure 5:**
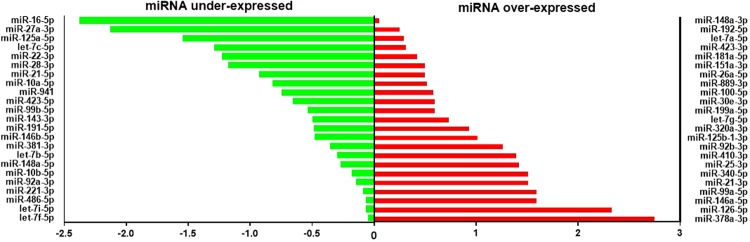
Differential expression of exosomal miRNA. Histograms of the human miRNome expression analysis depicting the distribution of differentially expressed miRNA in exosomes produced by AMSCs treated with or without 20 ng/ml IFNγ/TNFα. The graph was obtained by plotting the log_2_ fold change of normalized count of under-expressed (green) and over-expressed (red) miRNAs isolated from exosome released by treated cells.

Exosome-associated microRNAs were profiled using small RNA next generation sequencing, setting exosomes released by untreated cells as control samples and exosomes released by AMSCs treated with 20 ng/ml IFNγ/TNFα as test samples. The fold change was calculated dividing the normalized gene expression profile of test samples by the corresponding control samples. The activation with cytokines of AMSCs induced, in the released exosomes, the over-expression and the under-expression of 23 different miRNAs (Fig. 5 and Table 1).

**Table 1:** Loss and gain of miRNAs in exosomes produced by AMSCs treated with or without 20 ng/ml IFNγ/TNFα.

LOST miRNAs				GAINED miRNAs
let-7e-5p	miR-186-5p	miR-30a-3p	miR-671-3p	miR-100-3p
miR-125b-5p	miR-1910-5p	miR-335-3p	miR-7706	miR-101-3p
miR-134-5p	miR-193b-3p	miR-382-5p	miR-98-5p	miR-1246
miR-136-3p	miR-197-3p	miR-409-3p		miR-127-3p
miR-148b-3p	miR-19b-3p	miR-4677-3p		miR-155-5p
miR-150-5p	miR-23b-3p	miR-532-5p		miR-361-5p
miR-151a-5p	miR-27b-3p	miR-6515-5p		miR-411-5p
miR-181b-5p	miR-301a-3p	miR-654-5p		miR-493-3p

Next, we validated the RNA sequencing data focusing on specific miRNAs involved in the regulation of macrophage polarization^20^. By quantitative RT-PCR, we evaluated the expression of miRNAs regulating the differentiation towards M1 (miR-127-3p and miR-155-5p) or M2 (miR-34a-5p, miR124-3p, miR135b-5p and miR146a-5p) phenotypes. Of note, miR-21-5p is able to redirect both M1 and M2 polarization, depending on protein target.

**Figure 6:**
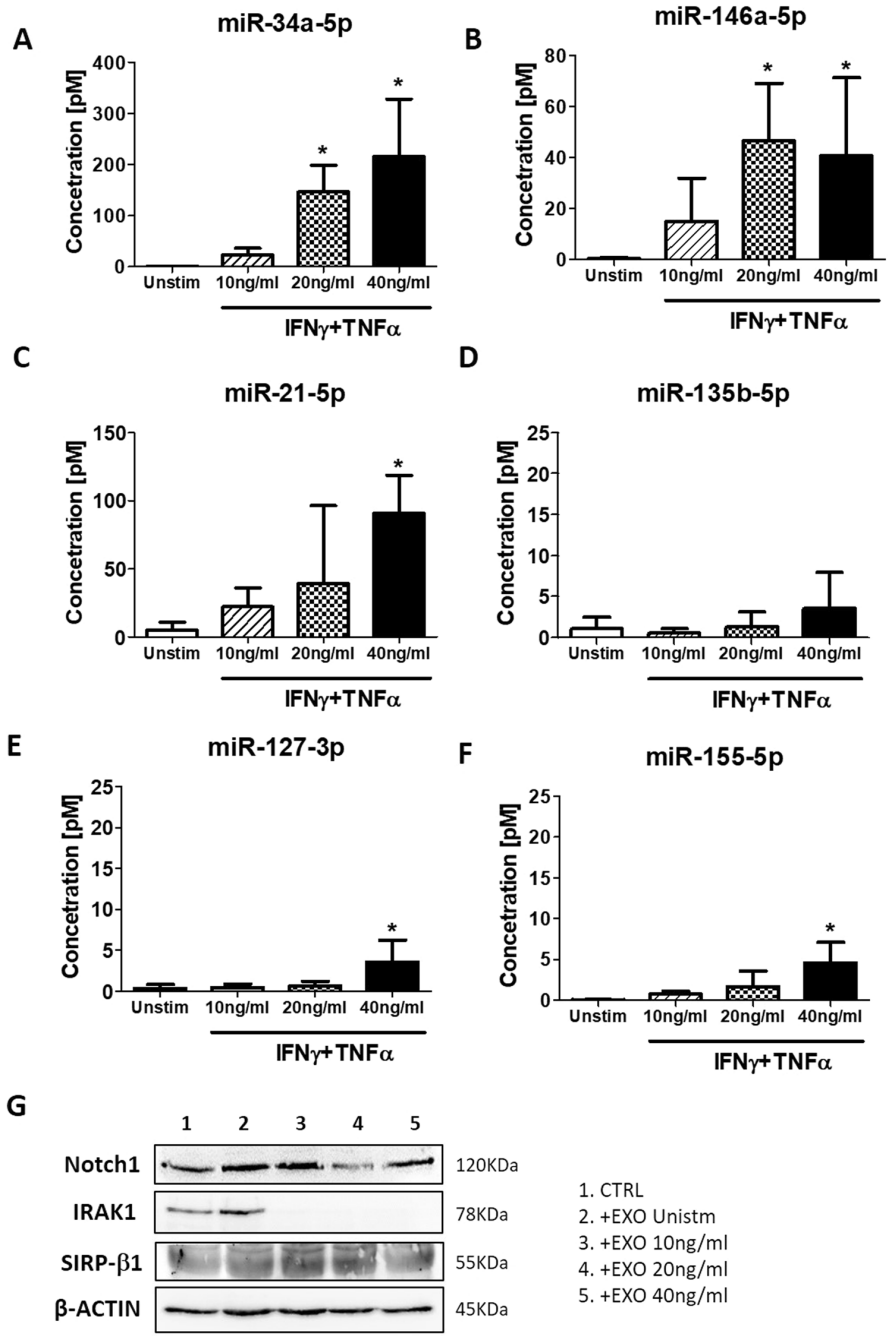
Exosomes derived from AMSCs pre-activated with inflammatory cytokines contained miRNA involved in M2 macrophages polarization. The concentration of miR-34 (**A**), miR-127 (**B**), miR-21 (**C**), miR-135 (**D**), miR-146 (**E**) and miR-155 (**F**) was measured in exosomes produced by AMSCs treated with or without 10, 20 and 40 ng/ml IFNγ/TNFα by qRT–PCR. Columns, mean; bars, SD, * significant difference from exosomes of unstimulated cells, *P* < 0.05. (**G**) Monocytes were differentiated in macrophages with GM-CSF in presence of exosomes isolated from the supernatants of unstimulated (EXO UNSTIM) or cytokines-activated (EXO IFNγ/TNFα 10, 20 and 40 ng/ml) AMSCs. Cell lysates were subjected to Western blot analysis with specific antibody against to IRAK1, Notch1, Sirp-β1 and β-actin (cropped images – uncropped originals available in Supplementary Figure 4).

All the miRNAs under investigation were expressed at low level in unstimulated AMSCs-derived exosomes (Fig. 6), except for miRNA-124-3p, which was undetectable (data not shown). The expression of miRNA-34 (Fig. 6A) and miRNA-146 (Fig. 6B) was significantly higher in exosomes produced by AMSCs pre-activated with 20 and 40 ng/ml IFNγ/TNFα compared to those of untreated cells, while miRNA-21 expression was significantly upregulated only for 40 ng/ml cytokine pre-stimulation (Fig. 6C). No difference was observed for the expression of miR-135 (Fig. 6D). The expression of miR-127 (Fig. 6E) and miR-155 (Fig. 6F) were significantly increased only in exosomes produced by AMSCs activated with the highest (40 ng/ml) cytokine concentration, but at a very lesser extent compared to the other described miRNAs.

Finally, to evaluate the downstream effect of miRNA expression upregulation in our AMSC experimental model, we choose to analyse the protein expression of some specific miRNA targets in macrophage lysates. In particular, we analysed the expression of Notch1, IRAK1 and Sirp-β1 targeted by miR-34a^21^, miR-146^22^ and miR-21^23^, respectively.

As illustrated in Fig. 6G, IRAK1 expression was dramatically reduced after treatment with exosomes of pre-stimulated AMSCs, while the expression of Notch1 was reduced only with exosomes release from cells treated with 20 ng/ml of cytokines. The expression of Sirp-β1 was not affected by the treatment with exosomes.

## Discussion

The unique MSCs immunosuppressive capability make them a promising therapeutic tool to suppress inflammation and to down-regulate the exaggerated immune response causing tissue damage by chronic inflammation or autoimmune disorders^3,24^. On the other hands, accumulating evidences support the notion that MSCs act mostly in a paracrine manner, through the release of soluble factors and extracellular vesicles, opening opportunities for secretome-based therapies, even if the mechanisms are not fully understood and the results remain controversial^14^.

The regenerative potential of MSCs-derived exosomes is a subject of fervent international investigation. In fact, a conclusive demonstration that exosomes may exert important immune-regulatory activities should open the way to the extended used of exosomes in cell-free regenerative medicine^25,26^. In this scenery, treatment with AMSCs-derived exosomes may have significant advantages, since they may replace live cells administration, mitigating many of the safety concerns and limitations associated with the transplantation of viable replicating cells.

In this study, we investigated the immunomodulatory properties of AMSCs-derived exosomes, discovering that an inflammatory stimulus activate AMSCs and can induce the release of exosomes with immunosuppressive abilities, since they are able to polarize macrophages towards the anti-inflammatory M2 phenotype. Our results suggest that the ability of AMSCs-derived exosomes to modulate the differentiation of macrophages is not constitutive, but rather activated by signals derived from a pro-inflammatory microenvironment.

There are evidences suggesting that MSCs produce immune modulatory and regenerative factors in response to inflammatory stimuli, in fact, it may be possible to enhance or suppress certain functions of MSCs by controlling their culture conditions, in particular, priming of MSCs with IFNγ and TNFα successfully improved their immunomodulatory functions^27^. Specifically, treatment with IFNγ up-regulated several genes involved in immunomodulation, such as HLA-DRA, CD274B7, IDO, VCAM1, ICAM2, and chemokines such as CCL8, CXCL9 and CXCL10^28^. In addition, an *in vitro* model showed that, after priming with TNFα plus IFNγ, MSCs were less potent at increasing cytokine production by activated PBMCs and more effective at inhibiting T-cell proliferation^29^. In our study, in agreement with previous data^27^, we demonstrated that AMSCs, stimulated with increasing concentrations of IFNγ and TNFα, release immunomodulatory factors.

We observed that, pre-treatment with pro-inflammatory cytokines induced AMSCs expression of IDO, which probably inhibits cells proliferation in an autocrine manner, as proposed by Croitoru-Lamoury *et al*.^5^. Moreover, we found that cytokines treatment induce the production of other immunomodulatory mediators, such as PGE_2_ and IL-10 as well as the chemokine CCL-2, which is critical for the chemotaxis of monocytes. It has been suggested that by this mechanism, chemokine recruits monocytes into close proximity to MSCs, whereupon the immunosuppressive factors produced locally, may influence the inflammatory process^30^.

Recently, it has been proposed that the immunomodulatory activity of MSCs is mediated by the synergism between secreted small molecules and exosomes^14,31,32^. In our study, we characterized AMSCs-derived exosomes, investigating in particular the influence of an inflammatory stimulus on their miRNA cargo and downstream functions. Of note, we demonstrated that only monocytes cultured in the presence of exosomes produced by cytokines-activated AMSCs, are able to transdifferentiate M1 pro-inflammatory to M2 anti-inflammatory macrophages.

It has been already documented that MSCs can educate macrophages to adapt to an anti-inflammatory and immune suppressive phenotype^33^ and can induce the differentiation of human M2 by direct and indirect contact mechanisms^34^, but little is known about the possible role of released exosomes and previous study on their immunosuppressive properties are controversial.

It has been reported that exosomes released from MSCs exert an inhibitory effect in differentiation and activation of T cells^16^ and activate monocytes to produce high levels of anti-inflammatory cytokines, finally polarizing activated CD4 + T cell into regulatory T cell^15^. On the other hands, it has been reported that extracellular vesicles produced by bone marrow- and adipose tissue-derived mesenchymal stromal cells failed to suppress lymphocyte proliferation^18^, suggesting that cell-cell contact may play an important role on the immunosuppressive potential mediated by MSCs. In the light of our data, it can be assumed that the discrepancy in literature data is probably due to the low/absent immunosuppressive potential of exosomes produced by unstimulated MSCs, since we demonstrated that such immunosuppressive effect is evident only when AMSCs are pre-activated by a pro-inflammatory stimulus.

Recently, it has been demonstrated that MSCs can release small RNAs via exosomes, which are increasingly implicated in intracellular communications^35^ and may participate in the resolution of chronic inflammation, enhancing the curative effects of MSCs^36^.

In order to explore whether AMSCs-derived exosomes exert their specific immunomodulatory effects through specific miRNAs, we evaluated the expression of miRNA involved in the regulation of macrophage polarization^20^, reporting an increase in the expression of miRNAs regulating the M2 phenotype (miR-34a-5p, miR21, miR146a-5p) in exosomes produced by pre-activated AMSCs compared to those released by untreated cells. In particular, miR-34 inhibits the transcription of pro-inflammatory cytokines targeting Notch1^21^, while miR-146 targets NF-kB signalling mediators such as IRAK1 and TRAF6 and promote the expression of M2-associated genes^37^. Regarding miR-21, there are controversial opinion on its role in mediating macrophage polarization. It has been proposed that in early stage of inflammation, pri-miR-21 exerts pro-inflammatory effects and, through STAT3 expression upregulation, polarizes macrophages towards the M1 phenotype^38^. By contrast, at the resolution phase, mature miR-21 regulates the anti-inflammatory response and polarize macrophages towards the M2 phenotype, suppressing Sirp-β1, that is a MEK/ERK_1/2_ pathway activator^23^. We also showed that exosomes produced by pre-activated AMSCs shuttled miR-146, which might participate in macrophage polarization toward M2 phenotype by targeting IRAK1. In line with our results, it has been hypothesized that miR-21, miR-146a and miR-181, which displayed the most significant and highest expression in MSCs-derived exosomes, could modulate the expression of pro-inflammatory genes, thus reducing or delaying inflammation and enhance wound healing^36^. Of note, it has been reported that miR-146a was strongly upregulated in exosomes released by MSCs stimulated with IL-1β and it contributes to mitigate inflammation and increase survival in septic mice^39^. Finally, it is possible to hypothesize that, in addition to our documented miRNA-mediated effect, other molecules carried by exosomes, could play a role in macrophages polarization. In particular, several studies have shown that exosomes derived from MSCs harbor cytokines and growth factors, such as TGFβ1, interleukin-6 (IL-6), IL-10, and hepatocyte growth factor (HGF), which have been shown to contribute to immunoregulation^40,41^.

In conclusion, the present study demonstrates that AMSCs release exosomes with measurable immunosuppressive effects only after pre-activation with pro-inflammatory cytokines, simulating an inflammatory microenvironment. As a matter of fact, exosomes released from pre-activated AMSCs show the capability to switch macrophages to M2-like phenotype by shuttling miRNAs regulating macrophages polarization. Our data strongly suggest that an inflammatory stimulus may be fundamental to induce the release of immunotherapeutic exosomes from AMSCs.

## Methods

### Adipose mesenchymal stem cells isolation and culture

Adipose mesenchymal stem cells (AMSCs) were isolated from adipose tissue obtained by lipoaspirates. A total number of six lipoaspirate samples were collected after informed written consent of donors. Ethical approval was obtained from the Medical Research Ethics Committee of the Centro di Riferimento Oncologico, IRCCS, Aviano, Italy (Consent CRO-2016-30).

Lipoaspirates were enzymatically dissociated using a 0.05% collagenase II solution for 20 minutes at 37 °C (Worthington) and, after neutralization of the enzyme, were centrifuged at 500 × *g* for 5 minutes and filtered through a 70 μm nylon mesh (Merck Millipore). Cells were seeded in minimum essential medium-α (MEM-α) supplemented with 10% FBS (Gibco), penicillin/streptomycin solution (10 mL/L), alanine/glutamine solution (2 mM), human epidermal growth factor (10 ng/ml), insulin solution (10 μg/ml), 2-fosfo-L-ascorbic acid, trisodium salt (100 μM) and dexamethasone (0.01 μM) (all from Sigma-Aldrich). Culture were kept at 37 °C, 5% CO_2_ and 95% humidity and cells were characterized by flow cytometry using MSCs positive markers (CD29, CD73, CD90 and CD105) and hematopoietic negative markers (CD34 and CD45) as described previously^42^. Cells were used for experiment between passage 2 and 5.

All methods of analysis were performed in accordance with the relevant guidelines and regulations with appropriate quality control.

### Activation of adipose mesenchymal stem cells with IFNγ and TNFα

To activate AMSCs with inflammatory factors, cells were seeded at density of 15,000 cells/cm^2^ and after 24 hours supernatant was replaced with fresh culture medium supplemented with 5% certified exosomes-free serum (Gibco) with recombinant human IFNγ and TNFα (Prepotech) at different concentrations (10, 20 and 40 ng/ml). The concentration 10 ng/ml corresponds to 200U/ml. After 48 hours, AMSCs were harvested and cell proliferation/viability was determined by trypan blue exclusion assay. For flow cytometry analysis, AMSCs were fixed and permeabilized with intracellular Fix/Perm solution (eBiosciences), incubated with FITC-conjugated indoleamine-pyrrole 2,3-dioxygenase (IDO) antibody (eBiosciences) for 15 min and then washed twice with PBS. Flow cytometry was carried out on the FACSCalibur (Becton Dickson) and data analysed using Flowing software. Supernatants were also harvested, centrifuged for 10 minutes at 14,000 × *g* and stored at −80 °C for exosomes isolation or cytokines detection. The concentration of IL-6, IL-10, IL-8 and CCL-2 was determined with a magnetic beads-based multiplex assay (Bio-plex Assay, Bio-Rad Laboratories), while prostaglandin E_2_ release (PGE_2_) was quantified with an enzyme-linked immunosorbent assay (ELISA) kit (Invitrogen).

### AMSCs-derived exosomes isolation and characterization

Exosomes were isolated from AMSCs supernatants by polymer precipitation methods with (ExoQuick-TC System Biosciences) as described previously^43^. The exosomes-containing pellet was resuspended in PBS buffer or lysis buffer for subsequent analysis. AMSCs-derived exosomes number was determined using the Exocet kit (System Biosciences), according to manufacturer’s instructions. Briefly, exosomes were lysed using a gentle lysis solution to preserve the enzymatic activity of the exosomal Acetylcholinesterase (AChE) enzyme. A standard curve was generated using known numbers of exosomes (as measured by NanoSight) and calibrated with a recombinant AChE enzyme standard solution provided in the kit.

The size distribution of the exosomes collected was determined using a qNano (Izon) nanopore-based exosome detection system, according to the manufacturer’s instructions.

AMSCs lysate, AMSCs-derived exosomes and Exoquick-derived supernatants were analysed for the expression of exosomal markers and contaminants by immunoblotting. Anti-CD9 (1:1000, System BioScience), anti-CD63 (1:1000, LS Bio), anti-CD81 (1:500, Abcam), anti-TSG101 (1:500, Abcam), anti-calnexin (1:1000 Enzo Life Technologies), anti-GRP94 (1:1000 Genetex) and anti-RISC (1:1000 Abcam) were used as primary antibodies. Horseradish peroxidase (HRP)-conjugated IgG antibody (1:1000, Dako) was used as the secondary antibody.

To ensure that IFNγ and TNFα were not present in our exosome preparation as contaminants, the culture medium supplemented with cytokines at different concentrations (10, 20 and 40 ng/ml) was incubated with Exoquick as described above and concentration of IFNγ and TNFα was measured by magnetic beads-based multiplex assay.

### Exosomal RNA isolation, library preparation/sequencing and RT-PCR

To preserve small RNAs, total RNA was extracted from 5 × 10^9^ AMSCs-derived exosomes, using mirVana PARIS Kit (Life Technologies), adding cel-miR39 spike-in as exogenous control (ThermoFisher Scientific). Extracted RNA quality and quantity was evaluated by NanoDrop™ 1000 Spectrophotometer (ThermoFisher Scientific) and was stored at −80 °C until use.

For miRNA profiling analysis, a pool of AMSCs-derived exosomes obtained in five different purification was examined. ‘TruSeq SmallRNA Sample Prep kit’ (Illumina) has been used for library preparation following the manufacturer’s instructions. Both RNA samples and final libraries were quantified by using the Qubit 2.0 Fluorometer (Invitrogen) and quality tested by Agilent 2100 Bioanalyzer RNA Nano assay (Agilent technologies). Libraries were then processed with Illumina cBot for cluster generation on the flowcell, following the manufacturer’s instructions and sequenced on single-end mode on NextSeq 500 (Illumina, San Diego, CA). The CASAVA 1.8.2 version of the Illumina pipeline was used to processed raw data for both format conversion and de-multiplexing.

The relative concentrations of miRNAs involved in regulation of macrophages M1 (has-miR-21-5p, has-miR-127-3p and has-miR-155-5p) and M2 (has-miR-34a-5p, has-miR124-3p, has-miR135b-5p and hsa-miR146a-5p) polarization were assessed using TaqMan® Advanced miRNA Assays (ThermoFisher Scientific), according to manufacturer’s instructions, except for cDNA templates that were diluted 1:2 instead of recommended 1:10.

Real-time reaction was performed on the Applied Biosystems QuantStudio 3 System. MiRNA relative concentrations were normalized using relative standard curve method obtained by serial dilutions of cel-miR39 (1nM-100fM).

### Monocytes isolation and differentiation into M1 macrophages

Human peripheral blood mononuclear cells (PBMCs) were isolated from EDTA-uncoagulated blood of blood donors by Ficoll gradient centrifugation (Millipore). Monocytes were separated from PBMCs by negative selection using a human CD14+ cell enrichment kit (StemCell Technologies) according to the manufacturer’s instructions and resuspended in RPMI medium supplemented with 10% heat inactivated fetal bovine serum (FBS), 1% glutamine, 1% pyruvate, 1% non-essential aminoacid, 1% penicillin/streptomycin, 1% Hepes (all from Euroclone). To remove the exosomal fraction present in FBS, serum was ultracentrifuged for 4 hours at 100,000 × *g*. Purity of monocytes was over 95% as judged by staining with anti-CD14 (eBiosciences) (data not shown).

For macrophages differentiation, CD14+ monocytes were seeded in multiwell plates at 5 × 10^5^/cm^2^ in complete RPMI medium supplemented with 100 ng/ml granulocyte macrophage-colony stimulating factor (GM-CSF, Peprotech) in presence of 8 × 10^8^ AMSC-derived exosomes; medium was changed completely every 3 days. On day 9, macrophages was harvested with TrypLE ^TM^ express detachment solution (Gibco) and characterized by flow cytometry for the expression of M1 and M2 macrophages markers using CD80, CD206 and CD163 antibodies (eBiosciences). Macrophages were also lysed in RIPA buffer and expression of IRAK1, Notch1 and SIRPb1 was analysed by immunoblotting. Anti-IRAK1 (1:1000, Cell Signalling), anti-β-actin (1:5000, Cell Signalling), anti-Notch1 (1:500, Cell signalling) and anti-Sirp-β1 (1:200, Santa Cruz Biotecnologies) were used as primary antibodies. Horseradish peroxidase (HRP)-conjugated IgG antibody (1:1000, Dako) was used as the secondary antibody.

### Statistical methods

Data are reported as mean ± standard deviation. Statistical analysis has been performed using GraphPad Software (version 7). Data were tested for normal distribution using the Kolmogorov-Smirnov test. Repeated measurements were analysed by one-way analysis of variance followed by the Bonferroni or Dunnett post test. *P* values < 0.05 was considered significant.

## Electronic supplementary material

Supplementary figures

## References

[1] Farini A, Sitzia C, Erratico S, Meregalli M, Torrente Y (2014). Clinical Applications of Mesenchymal Stem Cells in Chronic Diseases. Stem Cells Int..

[2] Glenn JD, Whartenby KA (2014). Mesenchymal stem cells: Emerging mechanisms of immunomodulation and therapy. World J. Stem Cells.

[3] Gao F (2016). Mesenchymal stem cells and immunomodulation: current status and future prospects. Cell Death Dis..

[4] Wang Y, Chen X, Cao W, Shi Y (2014). Plasticity of mesenchymal stem cells in immunomodulation: pathological and therapeutic implications. Nat. Immunol..

[5] Croitoru-Lamoury J (2011). Interferon-γ regulates the proliferation and differentiation of mesenchymal stem cells via activation of indoleamine 2,3 dioxygenase (IDO). PLoS One.

[6] Tu Z, Li Q, Bu H, Lin F (2010). Mesenchymal stem cells inhibit complement activation by secreting factor H. Stem Cells Dev..

[7] Noone C, Kihm A, English K, O’Dea S, Mahon BP (2013). IFN-γ stimulated human umbilical-tissue-derived cells potently suppress NK activation and resist NK-mediated cytotoxicity *in vitro*. Stem Cells Dev..

[8] Ryan JM, Barry F, Murphy JM, Mahon BP (2007). Interferon-gamma does not break, but promotes the immunosuppressive capacity of adult human mesenchymal stem cells. Clin. Exp. Immunol..

[9] Kwon YW (2013). Tumor necrosis factor-α-activated mesenchymal stem cells promote endothelial progenitor cell homing and angiogenesis. Biochim. Biophys. Acta.

[10] Doorn J, Moll G, Le Blanc K, van Blitterswijk C, de Boer J (2012). Therapeutic applications of mesenchymal stromal cells: paracrine effects and potential improvements. Tissue Eng. Part B. Rev..

[11] Timmers L (2011). Human mesenchymal stem cell-conditioned medium improves cardiac function following myocardial infarction. Stem Cell Res..

[12] Camussi G, Deregibus MC, Cantaluppi V (2013). Role of stem-cell-derived microvesicles in the paracrine action of stem cells: Figure 1. Biochem. Soc. Trans..

[13] Camussi G, Deregibus MC, Tetta C (2010). Paracrine/endocrine mechanism of stem cells on kidney repair: role of microvesicle-mediated transfer of genetic information. Curr. Opin. Nephrol. Hypertens..

[14] Yu B, Zhang X, Li X (2014). Exosomes derived from mesenchymal stem cells. Int. J. Mol. Sci..

[15] Zhang B (2014). Mesenchymal stem cells secrete immunologically active exosomes. Stem Cells Dev..

[16] Blazquez R (2014). Immunomodulatory Potential of Human Adipose Mesenchymal Stem Cells Derived Exosomes on *in vitro* Stimulated T Cells. Front. Immunol..

[17] Chen W (2016). Immunomodulatory effects of mesenchymal stromal cells-derived exosome. Immunol. Res..

[18] Gouveia de Andrade AV (2015). Extracellular vesicles secreted by bone marrow- and adipose tissue-derived mesenchymal stromal cells fail to suppress lymphocyte proliferation. Stem Cells Dev..

[19] McWhorter FY, Wang T, Nguyen P, Chung T, Liu WF (2013). Modulation of macrophage phenotype by cell shape. Proc. Natl. Acad. Sci..

[20] Essandoh K, Li Y, Huo J, Fan G-C (2016). MiRNA-Mediated Macrophage Polarization and its Potential Role in the Regulation of Inflammatory Response. SHOCK.

[21] Jiang P (2012). MiR-34a inhibits lipopolysaccharide-induced inflammatory response through targeting Notch1 in murine macrophages. Exp. Cell Res..

[22] Taganov KD, Boldin MP, Chang K-J, Baltimore D (2006). NF-kappaB-dependent induction of microRNA miR-146, an inhibitor targeted to signaling proteins of innate immune responses. Proc. Natl. Acad. Sci. USA.

[23] Caescu CI (2015). Colony stimulating factor-1 receptor signaling networks inhibit mouse macrophage inflammatory responses by induction of microRNA-21. Blood.

[24] Glenn JD, Whartenby KA (2014). Mesenchymal stem cells: Emerging mechanisms of immunomodulation and therapy. World J. Stem Cells.

[25] Rani S, Ryan AE, Griffin MD, Ritter T (2015). Mesenchymal Stem Cell-derived Extracellular Vesicles: Toward Cell-free Therapeutic Applications. Mol. Ther..

[26] Vishnubhatla, I., Corteling, R., Stevanato, L., Hicks, C. & Sinden, J. The Development of Stem Cell-derived Exosomes as a Cell-free Regenerative Medicine. *J*. *Circ*. *Biomarkers* 1 10.5772/58597 (2014).

[27] Madrigal M, Rao KS, Riordan NH (2014). A review of therapeutic effects of mesenchymal stem cell secretions and induction of secretory modification by different culture methods. J. Transl. Med..

[28] Lee MW (2015). Strategies to improve the immunosuppressive properties of human mesenchymal stem cells. Stem Cell Res. Ther..

[29] Cuerquis J (2014). Human mesenchymal stromal cells transiently increase cytokine production by activated T cells before suppressing T-cell proliferation: effect of interferon-γ and tumor necrosis factor-α stimulation. Cytotherapy.

[30] Ren G (2008). Mesenchymal Stem Cell-Mediated Immunosuppression Occurs via Concerted Action of Chemokines and Nitric Oxide. Cell Stem Cell.

[31] Pashoutan Sarvar D, Shamsasenjan K, Akbarzadehlaleh P (2016). Mesenchymal Stem Cell-Derived Exosomes: New Opportunity in Cell-Free Therapy. Adv. Pharm. Bull..

[32] Lai RC, Yeo RWY, Lim SK (2015). Mesenchymal stem cell exosomes. Semin. Cell Dev. Biol..

[33] Eggenhofer E, Hoogduijn MJ (2012). Mesenchymal stem cell-educated macrophages. Transplant. Res..

[34] Abumaree MH (2013). Human Placental Mesenchymal Stem Cells (pMSCs) Play a Role as Immune Suppressive Cells by Shifting Macrophage Differentiation from Inflammatory M1 to Anti-inflammatory M2 Macrophages. Stem Cell Rev. Reports.

[35] Baglio SR (2015). Human bone marrow- and adipose-mesenchymal stem cells secrete exosomes enriched in distinctive miRNA and tRNA species. Stem Cell Res. Ther..

[36] Ti D, Hao H, Fu X, Han W (2016). Mesenchymal stem cells-derived exosomal microRNAs contribute to wound inflammation. Sci. China. Life Sci..

[37] Vergadi E (2014). Akt2 deficiency protects from acute lung injury via alternative macrophage activation and miR-146a induction in mice. J. Immunol..

[38] Wang Z (2015). MicroRNA 21 Is a Homeostatic Regulator of Macrophage Polarization and Prevents Prostaglandin E2-Mediated M2 Generation. PLoS One.

[39] Song Y (2017). Exosomal miR-146a Contributes to the Enhanced Therapeutic Efficacy of Interleukin-1β-Primed Mesenchymal Stem Cells Against Sepsis. Stem Cells.

[40] Burrello J (2016). Stem Cell-Derived Extracellular Vesicles and Immune-Modulation. Front. cell Dev. Biol..

[41] Lai RC (2012). Proteolytic Potential of the MSC Exosome Proteome: Implications for an Exosome-Mediated Delivery of Therapeutic Proteasome. Int. J. Proteomics.

[42] Domenis R (2015). Adipose tissue derived stem cells: *in vitro* and *in vivo* analysis of a standard and three commercially available cell-assisted lipotransfer techniques. Stem Cell Res. Ther..

[43] Domenis R (2017). Systemic T Cells Immunosuppression of Glioma Stem Cell-Derived Exosomes Is Mediated by Monocytic Myeloid-Derived Suppressor Cells. PLoS One.

