# Supplementary material for: Pro inflammatory stimuli enhance the immunosuppressive functions of adipose mesenchymal stem cells-derived exosomes
Source: Sci Rep. 2018 Sep 6;8:13325. doi: 10.1038/s41598-018-31707-9 (PMC6127134; doi:10.1038/s41598-018-31707-9)
Supplement: Supplementary file 1 — Supplementary figures [file 41598_2018_31707_MOESM1_ESM.pdf]

SUPPLEMENTARY:

PRO INFLAMMATORY STIMULI ENHANCE THE IMMUNOSUPPRESSIVE FUNCTIONS OF ADIPOSE MESENCHYMAL STEM CELLS-DERIVED EXOSOMES

Rossana Domenis, Adriana Cifù, Sara Quaglia, Cinzia Pistis, Massimo Moretti, Annalisa Vicario, Pier Camillo Parodi, Martina Fabris, Kayvan R. Niazi, Patrick Soon-Shiong, Francesco Curcio

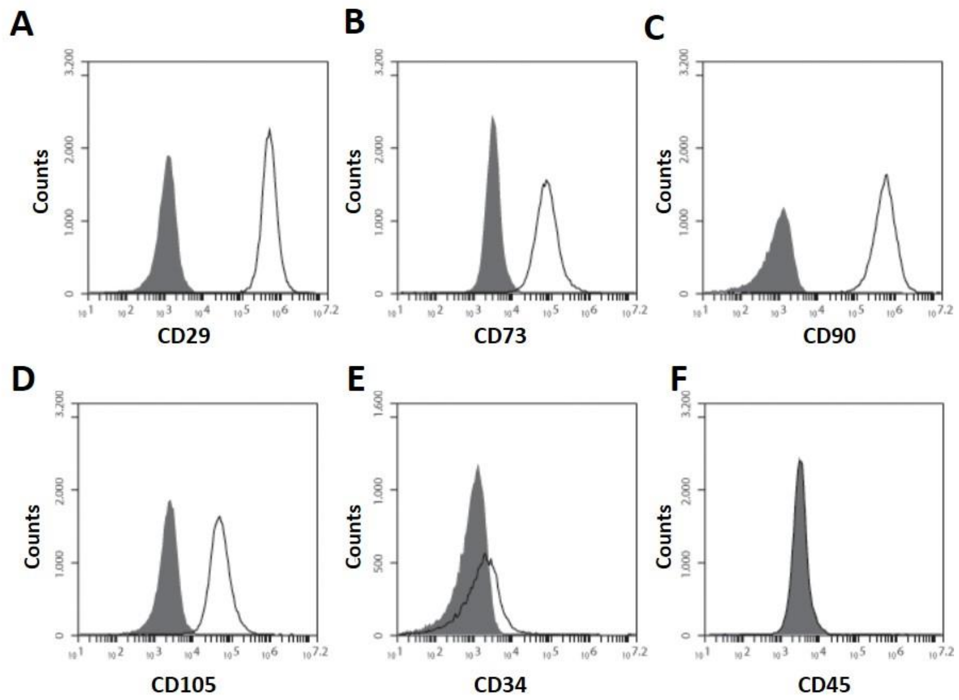

Supplementary Figure 1. Immunophenotype of AMSCs

Representative flow cytometry histograms of AMSCs stained for mesenchymal stem cell markers CD29 (A), CD73 (B), CD90 (D), CD105 (E) and hematopoietic markers CD34 (F) and CD45 (G). The antibodies (white peak) were compared with their appropriate isotype control (grey peak).

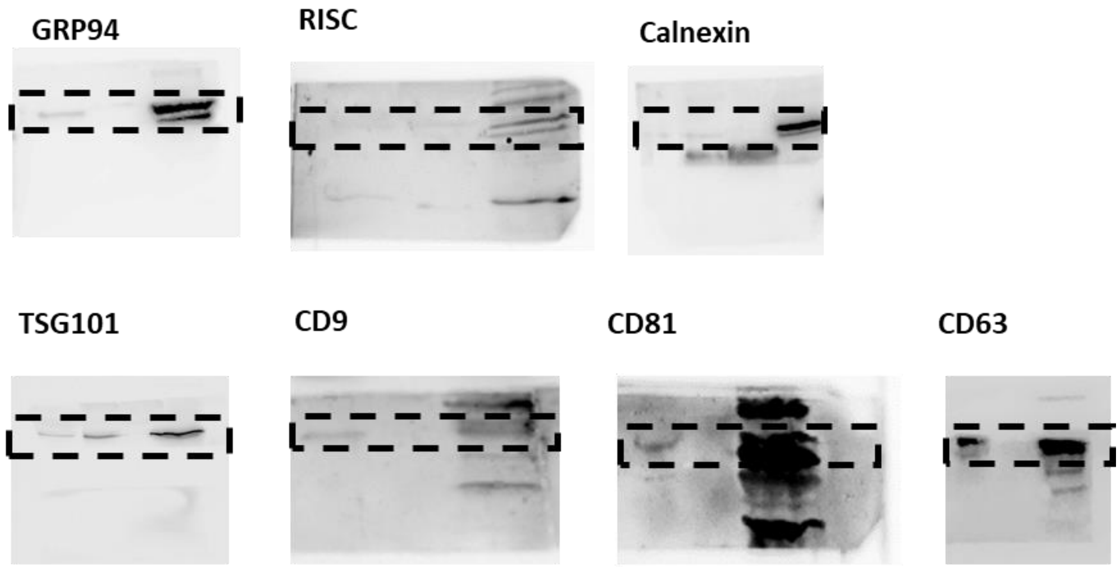

Supplementary Figure 2. Characterization of AMSCs-derived exosomes

Uncropped Western blots for, GRP94, RISC, Calnexin TSG101, CD9, CD81 and CD63.

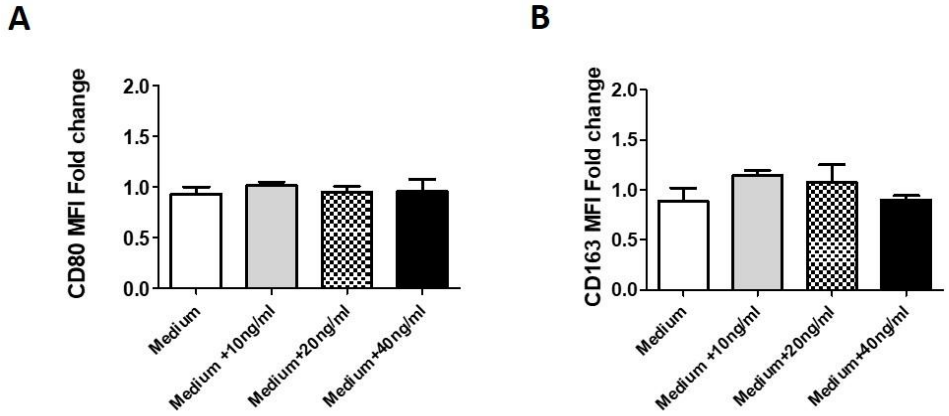

Supplementary Figure 3. Cytokines contamination from the culture medium did not affect macrophages polarization

Monocytes were differentiated into macrophages in presence of GM-CSF alone (CTRL) or in combination with cytokines contaminants isolated by polymer precipitation method from the culture medium supplemented with IFNγ/TNFα at different concentration (10, 20 and 40 ng/ml). Flow cytometry analysis of cell surface molecules CD80 (A) and CD163 (B) on macrophages. The levels of expression are presented as median fluorescent intensity (MFI) fold change respect untreated cells.

Notch1

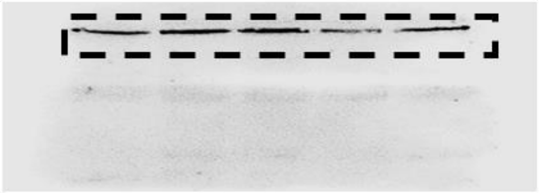

IRAK1 / β-ACTIN

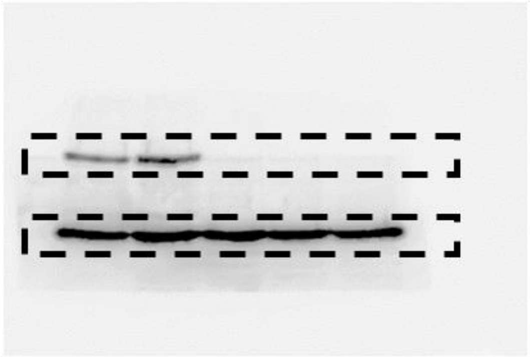

SIRP-β1

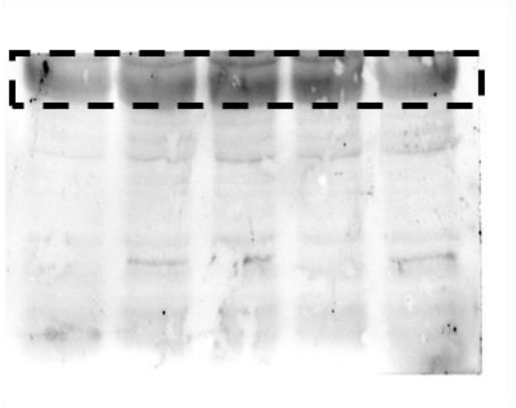

Supplementary Figure 4. Exosomes derived from AMSCs pre-activated with inflammatory cytokines contained miRNA involved in M2 macrophages polarization.

Uncropped Western blots for to IRAK1, Notch1, Sirp-β1 and β-actin
